# Application of Phase Congruency for Discriminating Some Lung Diseases Using Chest Radiograph

**DOI:** 10.1155/2015/424970

**Published:** 2015-03-31

**Authors:** Omar Mohd Rijal, Hossein Ebrahimian, Norliza Mohd Noor, Amran Hussin, Ashari Yunus, Aziah Ahmad Mahayiddin

**Affiliations:** ^1^Institute of Mathematical Science, University of Malaya, Lembah Pantai, 50603 Kuala Lumpur, Malaysia; ^2^UTM Razak School of Engineering and Advanced Technology, Universiti Teknologi Malaysia, UTM Kuala Lumpur Campus, Jalan Semarak, 54100 Kuala Lumpur, Malaysia; ^3^Institute of Respiratory Medicine, Kuala Lumpur Hospital, Jalan Pahang, 50590 Kuala Lumpur, Malaysia

## Abstract

A novel procedure using phase congruency is proposed for discriminating some lung disease using chest radiograph. Phase congruency provides information about transitions between adjacent pixels. Abrupt changes of phase congruency values between pixels may suggest a possible boundary or another feature that may be used for discrimination. This property of phase congruency may have potential for deciding between disease present and disease absent where the regions of infection on the images have no obvious shape, size, or configuration. Five texture measures calculated from phase congruency and Gabor were shown to be normally distributed. This gave good indicators of discrimination errors in the form of the probability of Type I Error (*δ*) and the probability of Type II Error (*β*). However, since 1 −  *δ* is the true positive fraction (TPF) and *β* is the false positive fraction (FPF), an ROC analysis was used to decide on the choice of texture measures. Given that features are normally distributed, for the discrimination between disease present and disease absent, energy, contrast, and homogeneity from phase congruency gave better results compared to those using Gabor. Similarly, for the more difficult problem of discriminating lobar pneumonia and lung cancer, entropy and homogeneity from phase congruency gave better results relative to Gabor.

## 1. Introduction

Lung cancer is the cause of more than a quarter of all cancer deaths in the United States and a major contributor of cancer death worldwide [1]. Despite efforts to control lung cancer, the chance of survival from this disease is still low (5–10% at five years). Annually 1.52 million new cases of lung cancer are detected and more than 1.31 million deaths are reported [2]. Lung cancer in early stages does not have clear signs and symptoms; however, continued coughing, shortness of breath, chest pains, and wheezing and coughing of blood are common indicators [3].

Pneumonia is a serious infection of the lungs and may have over 30 different causes. Symptoms of pneumonia include fever, coughing, shortness of breath, chest pain, and loss of appetite [4]. Over fifty-five thousand people died of pneumonia in the United States of America in the year 2006 [5]. Pneumonia is the ninth leading cause of death in 2010, with the highest mortality rate of all infectious diseases especially for people over 65 years of age [6].

Economic considerations create the situation where the digital chest X-ray is widely used [7]. For example, the Malaysian government hospitals perform a large number of diagnoses using radiograph films. However problems arise with the use of X-rays where studies have shown that the accuracy of the X-ray interpretation is subject to varying degrees of observer error [8]. Current methods or algorithms for disease detection mainly focus on the discrimination between normal images and images with signs of disease involving chest radiograph [9–15].

The study of image-based assessment of pulmonary infiltrates has seen the application of a wide variety of techniques; for example, Oliveira et al. [10] used wavelet, van Ginneken et al. [12] and Arzhaeva et al. [13] used texture, Katsuragawa and Doi [14] used contrast enhancement, Arzhaeva et al. [15] used two-class classifier, and Tsevas and Iakovidis used Gabor filter features [16].

Phase congruency (PC) features do not seem to be used for the assessment of the pulmonary infiltrates. Kovesi [17] showed that corners and edges are well detected using PC. The problem of discriminating between disease present and disease absent where the regions of infection on the images have no obvious shape, size, or configuration, sharp transitions between adjacent pixels strongly suggest using PC as a viable candidate for the development of features.

The use of PC was motivated by Oppenheim and Lim where phase information in a signal is more important than its amplitude [18]. Further developments involved the study of local energy model where the features are perceived at points in an image and the Fourier components are maximally in phase [19–23]. Venkatesh and Owens proved that local energy is proportional to phase congruency [24]. In 1999 Kovesi developed the phase congruency model based on log-Gabor transfer function for specific image applications [25].

One advantage of using PC is that it provides information about adjacent pixels. This property is particularly useful when the region of infection (ROI) does not conform to any standard shape, size, or configuration. Further the PC is invariant to changes in image brightness or contrast [25]. High congruency between phases (the value close to one) indicates large transition between adjacent pixel and low congruency between phases (the value close to zero) indicates mild transition between adjacent pixels [25]. Another advantage of PC is that it is a dimensionless measure and allows constant threshold values to be applied across wide classes of images. The properties of the phase congruency model have been used in new and interesting techniques in the recent decade to extract image features [17, 26–28].

Textures measures will be used as features for the discrimination. There are many techniques used in texture feature extraction. A popular technique is the use of wavelet textures; for example, in [9], Daubechies wavelet was applied. However, Daubechies wavelet does not preserve phase information [25]. Texture measures using cooccurrence matrix and markov random field parameters are sensitive to illumination and Gaussian noise [29], a problem which can be handled using PC. Multiresolution histogram does give texture information and in general they can discriminate between images with different spatial patterns alone without the help of other filters or features [30, 31]. Another technique that is widely used in capturing local structural patterns from images is Gabor filter [16]. References [32–38] reported that Gabor filters yield excellent texture descriptors. However in our study the region of infection does not exhibit any well- or easily defined spatial patterns. These remarks suggested that the potential of PC texture features should be explored.

The closest rival to the application of PC texture features is that of the application of Gabor texture features which will be compared in this study. Kovesi formulates PC using log Gabor as the filter function which maintains zero DC for arbitrarily large bandwidth compared to Gabor function (zero DC confined to one octave bandwidth only) [25]. Secondly log Gabor has extended tail at high frequency and therefore high frequency components are not suppressed resulting in preservation of image details [39, 40].

The discrimination ability of PC texture features relative to Gabor texture features in discriminating lobar pneumonia (PNEU) from normal healthy individuals (NL), lung cancer (LC) from NL, and between PNEU and LC using chest radiograph will be carried out. The texture measures are tested for normality. Existence of the univariate normal probability distributions allows optimal discrimination functions to estimate error probabilities, in particular, the probability of Type I Error (*δ*) and the probability of Type II Error (*β*). The final selection of texture measures was decided from the result of an ROC analysis.

## 2. Selection of Case Study

This study involved collaboration with the Institute of Respiratory Medicine (IPR), Malaysia, which is the national referral centre for respiratory diseases. The IPR provided archived patients' data which include chest X-ray films (blue-based films) captured using the Phillips Diagnost 55/Super 50CP (Phillips Corp., Holland) together with complete patients' medical information. The patient's chest was captured in full inspiration using the posterior-anterior (PA) view. The distance between the X-ray and the patient is fixed at 180 cm to diminish the effect of beam divergence and magnification of structures closer to the X-ray tube. The cassette sizes of 35 cm × 35 cm and 35 cm × 43 cm were used for female and male patients, respectively. A patient is exposed to 64 kV and 4.0 mAs if he or she is underweight, 70 kV and 5.0 mAs if he or she is normal weight, and 96 kV and 8 mAs if he or she is overweight (>100 kg). All overexposed and underexposed X-ray films as well as those with shadows are omitted from the study.

The archived data (stored in files) in IPR were diagnosed by a pulmonologist. In IPR, all the pulmonologists are trained to interpret chest radiographs. In this study, stratified random sampling (SRS) was carried out for the patients' files which were randomly selected given that the patients chosen were already diagnosed as LC or PNEU. The diagnoses of all cases were done by the IPR pulmonologists, and in this study the diagnoses were again verified by the consultant pulmonologist. It should be noted that the pulmonologist and consultant pulmonologist mentioned above are two different individuals. The LC cases consist of 75% cases of nonsmall cell carcinoma of which 50% of these are the squamous cell carcinoma and 25% are the adenocarcinoma. The other 25% cases are small cell carcinoma. The confirmation of LC was based on bronchial biopsy result. The confirmation of PNEU cases was based on chest X-ray findings and sputum culture test. This sputum test is done to identify the causative organism, and in this study, the commonest organism isolated is* Streptococcus pneumoniae* bacteria, while the chest X-ray is to identify the type and site of infection. The commonest type noted in this study is lobar pneumonia. The selected patients used were the confirmed LC and PNEU cases with no other systemic diseases such as diabetes, hypertension, and heart disease. The omission of cases, with the help of the consultant pulmonologist, with other systemic diseases was done in order to avoid bias in the development of a statistical discriminant function. The normal lung (NL) chest X-ray films selected by the radiologist from IPR represent patients who came for a general medical check-up. The complete dataset consist of 40 NL, 50 LC, and 50 PNEU cases.

**Figure 1:**
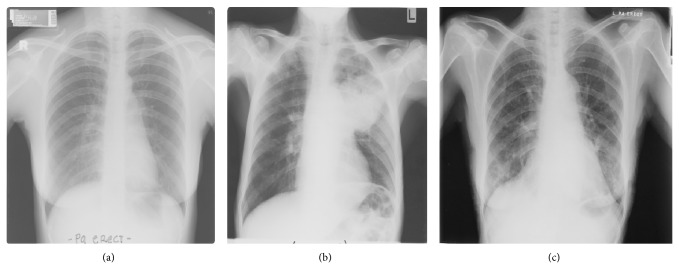
Digital chest X-rays of (a) normal lung, (b) LC in left upper zone, and (c) PNEU in right and left lower zone (source: Insitute of Respiratory Medicine, Kuala Lumpur).

The chest X-ray films were then digitized into DICOM format using the Kodak LS 75 X-ray Film Scanner (pixel spot size of 100 *µ*m, 12 bit per pixel, and image size of 2016 × 2048 pixels). Examples of a digitized X-ray film for normal lung and infected lung with LC and PNEU are shown in Figure 1. All digital image X-rays underwent normalization process as described in the next section.

### 2.1. Normalization of the Chest Radiograph

Image normalization was done on the whole dataset to make image intensities comparable across the whole dataset [16]. LC or PNEU cases will have their chest X-ray film image exhibitting some abnormal opacity. The landmarks on the spine were selected following [16] where these landmarks remain practically unaffected by the presence of infiltrates. Other than the spine, other lung anatomies especially the soft tissues are not suitable to become normalization landmark since it will be affected by the presence of infiltrates. A landmark, for example, the seventh cervical vertebra (C7), was selected for each patient in a given category (example NL) and the corresponding pixel intensity at the centre of C7 was studied. This process was repeated for other landmarks, 1st–4th thoracic vertebrae (*T*1, *T*2, *T*3, and *T*4, resp.), all of which were taken at the center of the spine. The summary statistics illustrated in Tables 1(a), 1(b), and 1(c) showed that the variation of the intensity is small.

To eliminate problem the normalization of the chest radiograph was done on the dataset as proposed by [16].

**Figure 2:**
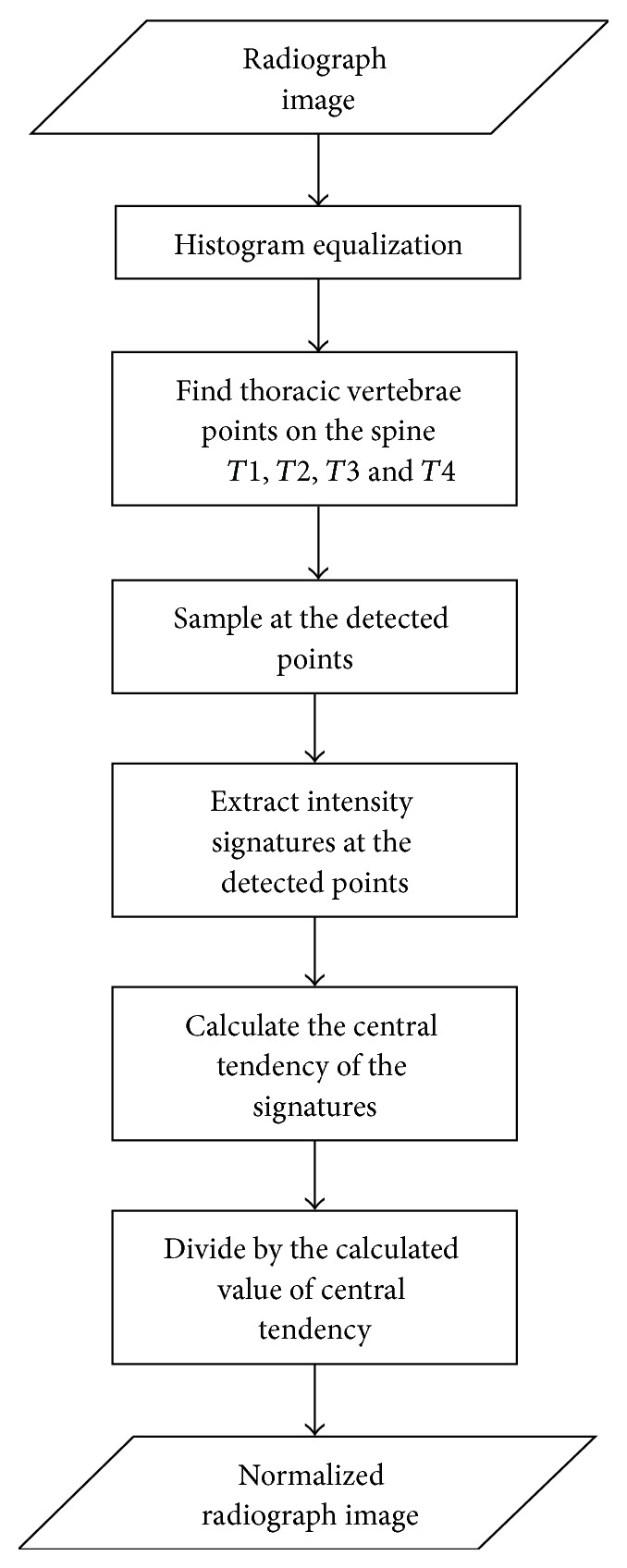
Chest radiograph normalization process following method in [16].

In order to enhance the discrimination procedure X-ray images were preprocessed by histogram equalization and image normalization processes.  Figure 2 illustrates the normalization procedure proposed [16]. The procedure is as follows.

**Figure 3:**
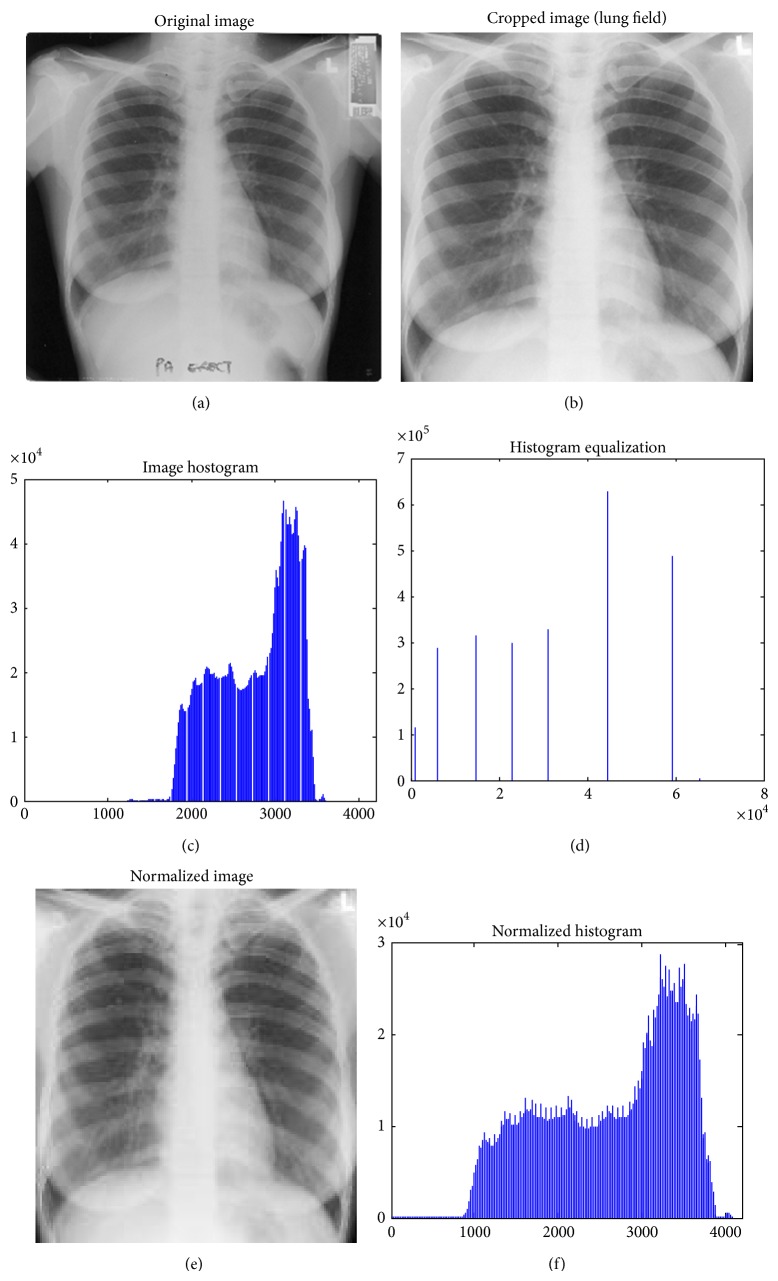
Images of normalization procedure.

(1) The original images were cropped in order to have only the lung fields.
(2) Plot the image histogram of each image.
(3) In order to have uniform distribution of intensities, for each resultant image the histogram equalization procedure was implemented.
(4) Four landmarks, namely, the middle of 1st–4th thoracic vertebrae (*T*1, *T*2, *T*3, and *T*4 resp.), are selected as references.
(5) Steps 1 to 4 were repeated for all 40 NL, 50 LC, and 50 PNEU cases.
(6) Let $X=[\bar{T1},\bar{T2},\bar{T3},\bar{T4}]$ be the central tendency of the intensity references at selected landmarks. Let
  (1) $$\begin{array}{c} S1={\left[\frac{T1_{i}}{\bar{T1}}\right]}^{-1},\;\;S2={\left[\frac{T2_{i}}{\bar{T2}}\right]}^{-1},\\ S3={\left[\frac{T3_{i}}{\bar{T3}}\right]}^{-1},\;\;S4={\left[\frac{T4_{i}}{\bar{T4}}\right]}^{-1}, \end{array}$$
 where *i* = 1,…, 140 (40 NL, 50 LC and 50 PNEU).
 Let $\text{CS}=\left(\sum_{i=1}^{4}{\bar{S1}}_{i}\right)/4$ be the central signature of our data base.
(7) Let *I* be a given radiograph. The image was then normalized by dividing the image intensities with the central signature, *I*_*n*_ = *I*/CS, where *I*_*n*_ is the resultant normalized image.  Figure 3 shows the original image and the normalized image and its respective histogram.

## 3. Phase Congruency Model

The two-dimensional phase congruency model for any pixel *x* is given as follows [25]:

(2) $$\begin{array}{c} \text{PC}\left(x\right)=\frac{\sum_{o}\sum_{n}W_{o}\left(x\right)\left\lfloor A_{no}\left(x\right)\Delta \Phi_{no}\left(x\right)-T_{o}\right\rfloor }{\sum_{o}\sum_{n}A_{no}\left(x\right)+\varepsilon }, \end{array}$$

such that if the value of *A*_*no*_(*x*)ΔΦ_*no*_(*x*) − *T* is negative, the function ⌊·⌋ returns a value zero; otherwise, it returns its arguments.

The component *W*_*o*_(*x*) = 1/(1 + *e* ^*γ*(*c*−*s*(*x*))^) is the weighting function at orientation *o*, where

(3) $$\begin{array}{c} s\left(x\right)=\frac{1}{N}\left(\frac{\sum_{n}A_{n}\left(x\right)}{\varepsilon +A_{\max}\left(x\right)}\right), \end{array}$$

and *N* is the total number of scale being considered, *ε* is a small positive number added to prevent division by zero, *A*_max⁡_ is the amplitude of the filter pair having maximum response at pixel *x*, *γ* is the gain factor to control the sharpness of cut-off filter, *c* is the cut-off filter value, and *A*_*n*_ is the amplitude of the transform at the given wavelet scale *n* using log-Gabor filter,

(4) $$\begin{array}{c} G\left(w\right)=\exp\left(\frac{-{\left(\log\left(w/w_{0}\right)\right)}^{2}}{2{\left(\log\left(\kappa /w_{0}\right)\right)}^{2}}\right), \end{array}$$

where *w* is the angular frequency, *w*_0_ is the filter's centre frequency, and the term *σ* = *κ*/*w*_0_ = 0.55 gives approximately two-octave bandwidth. *w*_*o*_ = 1/*λ*_max⁡_, where *λ*_max⁡_ = *λ*_min⁡_ · *α* ^*n*−1^, and *α* is the scaling between centre frequencies of successive filters. The wavelength of the smallest scale is denoted as *λ*_min⁡_. The angular overlap of the filter transfer function is controlled by the ratio of the angular interval between filter orientations and the standard deviation of the angular Gaussian spreading function (denoted by *d*).

A measure of angular phase deviation that is approximately linear is given by

(5) $$\begin{array}{c} \Delta \Phi_{no}\left(x\right)=\cos\left(\phi_{no}\left(x\right)-\overset{-}{\phi }\left(x\right)\right)-\left|\sin\left(\phi_{no}\left(x\right)-\overset{-}{\phi }\left(x\right)\right)\right|, \end{array}$$

where *ϕ* and $\bar{\phi }$ are the local phase and the mean phase angle, respectively. *T*_*o*_ = *μ*_*R*_ + *kσ*_*R*_ compensates the effect of noise at orientation *o*, where *μ*_*R*_ and *σ*_*R*_^2^ are the mean and variance of the Rayleigh distribution of the noise energy response and *k* = constant (typically 2 or 3). *A*_*no*_(*x*) is the amplitude of the transform at the given wavelet scale *n* and orientation *o*.

## 4. Gabor Model

Gabor's theory implies that the information can be represented by the amplitudes of functions that are localized in both space and frequency [39]. By using 2D Gabor filter, orientation and scale information can be captured.

Gabor filtered image is obtained by convolving image *I*(*x*, *y*) with a two-dimensional Gabor function *g*(*x*, *y*) given by [41] as follows:

(6) $$\begin{array}{c} r\left(x,y\right)=\left\lfloor \overset{}{\underset{\Omega }{\iint }}I\left(x,y\right)g\left(x,y\right)dx\;dy\right\rfloor , \end{array}$$

where ⌊*z*⌋ returns the value zero if *z* is negative; otherwise, it returns its arguments. Two-dimensional Gabor function, *g*(*x*, *y*), is given as

(7) $$\begin{array}{c} g_{\xi ,\eta ,\lambda^{*},\Theta ,\varphi }\left(x,y\right)=\exp\left(-\frac{x^{\prime 2}+\gamma^{*2}y^{\prime 2}}{2\sigma^{*2}}\right)\cos\left(2\pi \frac{x^{\prime }}{\lambda^{*}}+\varphi \right),\\ x^{\prime }=\left(x-\xi \right)\cos\Theta -\left(y-\eta \right)\sin\Theta ,\\ y^{\prime }=\left(x-\xi \right)\sin\Theta -\left(y-\eta \right)\cos\Theta , \end{array}$$

where the arguments *x* and *y* are the position of a light impulse in the visual field, (*ξ*, *η*) is the center of a receptive field, *σ* ^∗^ is the standard deviation of the Gaussian factor that determines the size of the receptive field, *γ* ^∗^ is the spatial aspect ratio (in this study we use *γ* ^∗^ = 0.5), *λ* ^∗^ is the wavelength from the cosine factor cos⁡⁡(2*π*(*x*′/*λ* ^∗^) + *φ*) such that *σ* ^∗^/*λ* ^∗^ is the spatial frequency bandwidth, Θ  (Θ ∈ [0, *π*)) is the orientation of the filter, and *φ* is the phase offset that determines the symmetry of the *g*(*x*, *y*).

## 5. Methods

Three random samples consisting of 40 NL, 50 LC, and 50 PNEU cases were used in this study. The consultant pulmonologist determined the ROI as ground truths. The PC parameters were then determined in a simulation study. It should be noted that the Gabor model is chosen in such a way that it has the same number of channels and similar channel characteristic as in the PC model. The ROI size for both model is fixed at 256 × 256 pixels. Once the parameter values are selected, the corresponding texture measures were calculated. Summary statistics of texture measures investigate the range of values of the texture measures. Test of normality on texture measures in turn indicates the suitability of texture measures to be used in an optimal statistical discrimination procedure. Finally an ROC analysis determined the best choice of texture measures and this enables a comparison between PC and Gabor features in discriminating between PNEU and NL and between LC and NL. A comparison was also made in discriminating between PNEU and LC.

### 5.1. Selecting Phase Congruency Parameter Values

**Figure 4:**
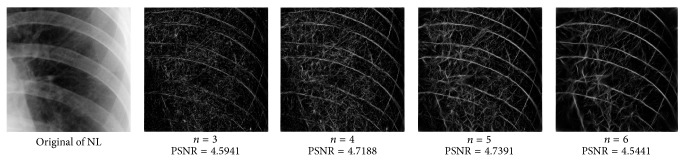
Varying of wavelet scale numbers, *n*.

**Figure 5:**
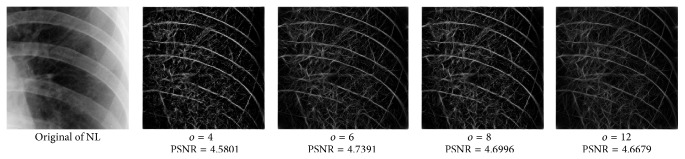
Varying *o* with *n* = 5.

**Figure 6:**
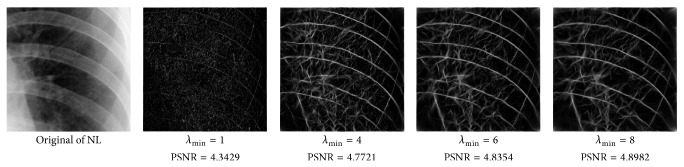
Varying *λ*_min⁡_ with *n* = 5, *o* = 6.

**Figure 7:**
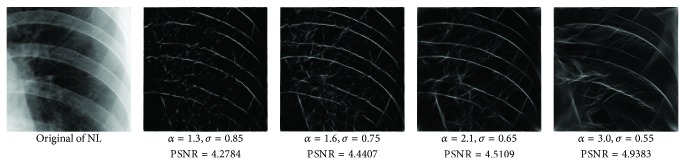
Varying *α* and *σ* with *n* = 5, *o* = 6, and *λ*_min⁡_ = 8.

**Figure 8:**
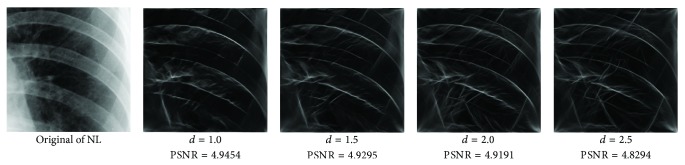
Varying *d* with *n* = 5, *o* = 6, *λ*_min⁡_ = 8, *α* = 3, and *σ* = 0.55.

**Figure 9:**
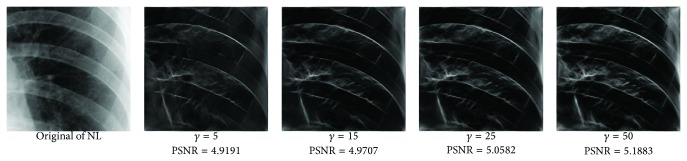
Varying *γ* with *n* = 5, *o* = 6, *λ*_min⁡_ = 8, *α* = 3, *σ* = 0.55, and *d* = 1.

**Figure 10:**
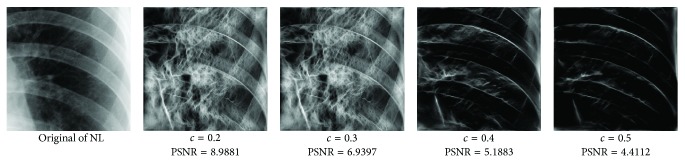
Varying *c* with *n* = 5, *o* = 6, *λ*_min⁡_ = 8, *α* = 3, *σ* = 0.55, *d* = 1, and *γ* = 50.

**Figure 11:**
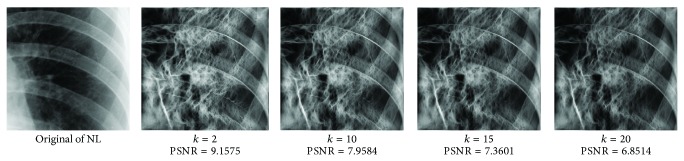
Varying *k* with *n* = 5, *o* = 6, *λ*_min⁡_ = 8, *α* = 3, *σ* = 0.55, *d* = 1, *γ* = 50, and *c* = 0.2.

A simulation study was carried out to find optimal values for the parameters *n*, *o*, *λ*_min⁡_, *α*, *σ*, *d*, *γ*, *c*, and *k* of (2) using the NL images (ROI size fixed at 256 × 256 pixels). The first estimate of the parameters is the default values suggested by Kovesi [25] using *n* = 3, *o* = 6, *λ*_min⁡_ = 3, *α* = 2, *σ* = 0.55, *d* = 1.7, *γ* = 10, *c* = 0.4, and *k* = 3, which does not give clear distinction between the rib-bones and lung tissue. The parameter values are varied as follows: Figure 4 shows the effect of varying *n* while keeping the other default values fixed. For each value of *n*, the power signal to noise ratio (PSNR) value between the original image and transformed image was calculated and clearly the case *n* = 5 gave the highest PSNR value (PSNR = 4.7391). Similarly, varying *o* with *n* = 5 and all remaining parameters at their default values suggests selecting *o* = 6, PSNR = 4.7391 (Figure 5). This process is continued, as illustrated in Figures 6, 7, 8, 9, 10, and 11 until the combination of *n* = 5, *o* = 6, *λ*_min⁡_ = 8, *α* = 3, *σ* = 0.55, *d* = 1, *γ* = 50, *c* = 0.3, and *k* = 2 yielded the highest PSNR value (9.1575). These optimal PC parameters found for the NL case were then applied to the LC and PNEU case (see Table 2).

The consultant pulmonologist studied these images in comparison with the original image and was able to locate the ROI. To further investigate the suitability of the parameter values, a selected line profile which is a plot of PC(*x*) values versus pixel position was obtained and shown in Table 2. The corresponding 2D profile is also given. The 1-D line profile gave clear differences but is less obvious for the 2D profile which gives motivation for using PC(*x*) values to discriminate LC from PNEU. Texture measures of PC(*x*) values may in turn be possible candidates for features in a given discrimination procedure.

### 5.2. Gabor Filter Parameters

To enable reasonable comparison between PC and Gabor, in this study we use the same number of channels and similar channel characteristics. The number of channels is 30 (wavelet scale *n* = 5, orientation *o* = 6). The other parameters used for Gabor filter are *σ* ^∗^/*λ* ^∗^ = 0.31 and *γ* ^∗^ = 0.5, and phase offset, *φ* = −*π*/2. The ratio *σ* ^∗^/*λ* ^∗^ determines the spatial frequency bandwidth, and since PC model used two-octave bandwidth, the same bandwidth size is utilized for Gabor model. The ratio *σ* ^∗^/*λ* ^∗^ = 0.31 gives two-octave bandwidth [42]. *γ* ^∗^ is the spatial aspect ratio (in this study we use *γ* ^∗^ = 0.5). The spatial aspect ratio has been found to vary in a limited range of 0.23 < *γ* ^∗^ < 0.92 [43]. The values *γ* ^∗^ = 0.5 and *φ* = −*π*/2 are used in our study as suggested by [42]. The size of ROI is fixed at 256 × 256 pixels.

### 5.3. Phase Congruency and Texture Measures

The PC(*x*) values were obtained for the selected ROI (fixed at 256 × 256 pixels), and subsequently a texture measure may then be calculated. There are many possible texture measures that could be used; however, in this study energy (*E*), mean energy ($\bar{E}$), entropy (En), contrast (*C*), homogeneity (*H*), standard deviation of value (STDV), standard deviation of energy (STDE), and correlation (Corr) texture measures [11] are considered. The mean and standard deviations of texture measures using PC model are listed in Table 3 and the corresponding table for Gabor model is given in Table 4. A comparison of these two tables shows that texture measures that tend to be better estimated using PC.

### 5.4. A Test of Normality

Amongst the many tests for univariate normality the Kolmogorov-Smirnov test is a widely used test that has high power even for relatively small sample size (less than 30). The Kolmogrov-Smirnov test statistic is *K*-*stat * = max⁡(|*F*(*X*) − *G*(*X*)|), where *F*(*X*) and *G*(*X*) are the empirical and assumed normal cumulative distribution function (CDF) of the random variable *X*, respectively. The hypothesis of normality is rejected when the value of *K*-stat is greater than the selected critical value [44].

**Figure 12:**
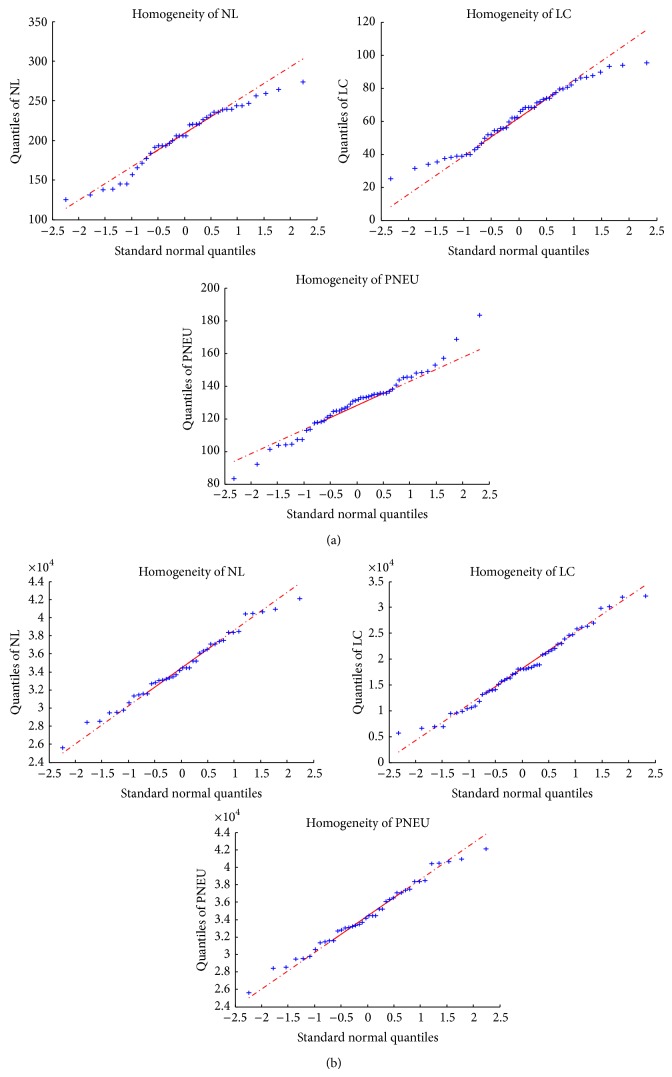
QQ plots for (a) homogeneity PC texture measures for NL, LC, and PNEU, respectively, and (b) homogeneity Gabor texture measures for NL, LC, and PNEU, respectively.

In total 140 images were used in this study where seventy images were used as control group in which 20 are from NL, 25 from LC, and 25 from PNEU. The other seventy images comprised of 20 NL, 25 LC, and 25 PNEU are used as the test group. Normality was tested at a 95% significance level. Homogeneity, energy, mean energy, entropy, and contrast were shown to be normally distributed for both PC and Gabor models.  Table 5 illustrates the test results for homogeneity using PC model and Gabor model. The results of normality testing were graphically verified by QQ plots as shown in Figure 12.

### 5.5. Statistical Discrimination

Without loss of generality the discrimination problem for LC and NL will be discussed following the notations of Johnson and Wichern [45]. Since the selected texture measures are normally distributed, an optimal discrimination procedure can be developed using *Q*(*x*) = ln⁡⁡(*f*_LC_(*x*)/*f*_NL_(*x*)), where *f*_LC_(*x*) and *f*_NL_(*x*) represent the probability density functions for LC and NL, respectively. When the sample data from both populations have equal variances, we use the well-known linear discriminant function (LDF), $\hat{Q}\left(x_{0}\right)={\left({\overset{-}{x}}_{\text{LC}}-{\overset{-}{x}}_{\text{NL}}\right)}^{T}S^{-1}x_{0}-\left(1/2\right){\left({\overset{-}{x}}_{\text{LC}}-{\overset{-}{x}}_{\text{NL}}\right)}^{T}S^{-1}\left({\overset{-}{x}}_{\text{LC}}+{\overset{-}{x}}_{\text{NL}}\right)$; otherwise the quadratic discriminant function (QDF), $\hat{Q}\left(x_{0}\right)=-\left(1/2\right)x_{0}^{T}(S_{\text{LC}}^{-1}-S_{\text{NL}}^{-1})x_{0}+({\overset{-}{x}}_{\text{LC}}^{T}S_{\text{LC}}^{-1}-{\overset{-}{x}}_{\text{NL}}^{T}S_{\text{NL}}^{-1})x_{0}-k$, where $k=\left(1/2\right)\ln(\left|S_{\text{LC}}\right|/\left|S_{\text{NL}}\right|)+\left(1/2\right)({\overset{-}{x}}_{\text{LC}}^{T}S_{\text{LC}}^{-1}{\overset{-}{x}}_{\text{LC}}-{\overset{-}{x}}_{\text{NL}}^{T}S_{\text{NL}}^{-1}{\overset{-}{x}}_{\text{NL}})$ will be used.

If $\hat{Q}\left(x_{0}\right)\geq \ln(C)$, then *x*_0_ is identified as LC; otherwise, *x*_0_ is identified as NL, where *C* = [(*c*(LC∣NL)/*c*(NL∣LC)) · (*p*_NL_/*p*_LC_)], ${\overset{-}{x}}_{\text{LC}}=(1/n_{\text{LC}})\sum_{i=1}^{n_{\text{LC}}}x_{\text{LC}}$, $S_{\text{LC}}=(1/\left(n_{\text{LC}}-1\right))\sum_{i=1}^{n_{\text{LC}}}{\left(x_{\text{LC}}-{\overset{-}{x}}_{\text{LC}}\right)}^{2}$, ${\overset{-}{x}}_{\text{NL}}=(1/n_{\text{NL}})\sum_{i=1}^{n_{\text{NL}}}x_{\text{NL}}$, and $S_{\text{NL}}=(1/\left(n_{\text{NL}}-1\right))\sum_{i=1}^{n_{\text{NL}}}{\left(x_{\text{NL}}-{\overset{-}{x}}_{\text{NL}}\right)}^{2}$ given that *n*_LC_ and *n*_NL_ denote the size of the control dataset from LC and NL, respectively. Finally,

(8) S=nLC−1nLC−1+nNL−1SLC+nNL−1nLC−1+nNL−1SNL.

The choice of LDF and QDF follows the result of testing the hypothesis *H*_0_ : *σ*_1_^2^ = *σ*_2_^2^, where *σ*_*j*_^2^ is the variance of *x*_*j*_ *j* = 1,2.

Twenty-five LC cases and twenty NL cases were used to estimate the parameters for a given texture measure TM. The remaining dataset was used as the test sample.

Let the null hypothesis be *H*_0_ : TM shows LC and, alternatively, *H*_1_ : TM shows NL. Type I Error occurs when the null hypothesis is rejected incorrectly and Type II Error occurs when the null hypothesis is accepted incorrectly. Henceforth the probability of Type I Error and Type II Error is as follows: *δ*1 = *P*(reject  *H*_0_∣*H*_0_ true) = *P*(NL∣LC) and *β*1 = *P*(accept  *H*_0_∣*H*_0_ false) = *P*(LC∣NL).

Similarly *δ*2, *β*2, and *δ*3, *β*3 are estimated corresponding to the pairs (PNEU, NL) and (LC, PNEU), respectively. A similar procedure was applied using Gabor features. Results are shown in Table 6 for all five texture measures that are normally distributed.

## 6. Receiver Operating Characteristic (ROC) Analysis

The performance of the discrimination procedure is indicated in terms of the size of *δ* and *β* for equal cost of misclassifications and equal a priori probabilities (*C* = 1); see Table 6. In practice this assumption does not hold and instead all possible combinations of these parameters should be considered for a given texture measure. This problem was solved by calculating *δ* and *β* for all possible values of *C* = (*c*(1∣2)/*c*(2∣1))(*p*_2_/*p*_1_) followed by the calculation of the true positive fraction (TPF) and the false positive fraction (FPF).

**Figure 13:**
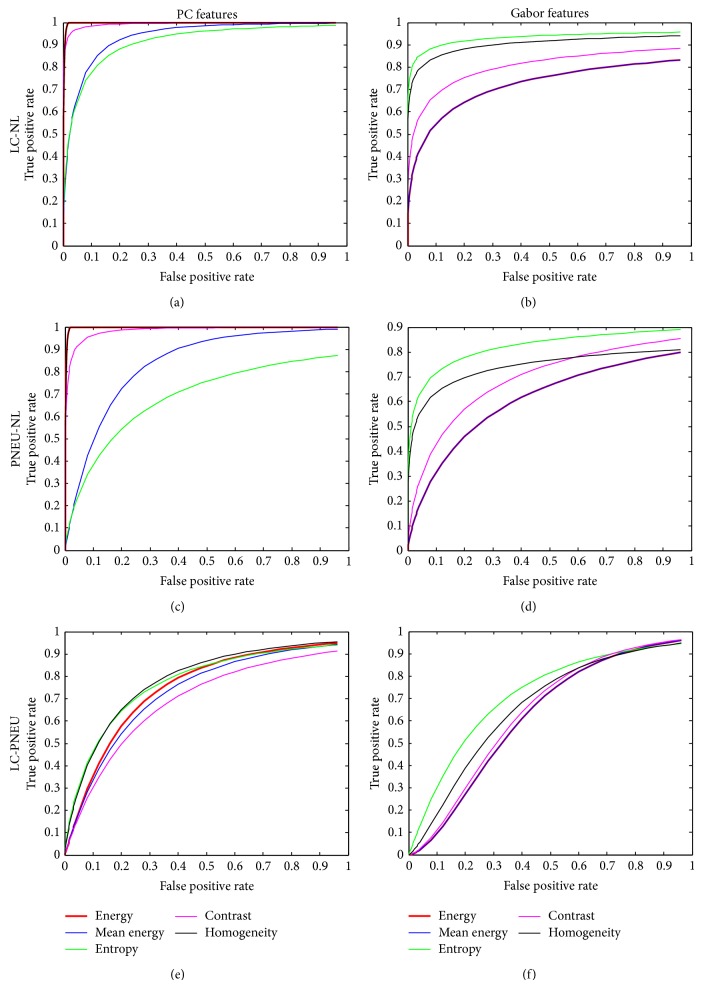
The ROC curves for discriminating: (a), (b) LC and NL, (c), (d) PNEU and NL, and (e), (f) LC and PNEU using PC and Gabor features, respectively.

Clearly TPF equals 1 − *δ* and FPF equals *β*. A plot of FPF and TPF for increasing values of *C* greater than zero is defined as an ROC curve for the texture measure considered. The choice of two texture measures is made by comparing their corresponding ROC curves (see Figure 13). The ROC curve which is left most and highest will imply that the corresponding texture measure should be preferred.  Table 7 shows the area under the ROC curve (AUC) for each texture measure.

## 7. Results and Discussion

The chest X-ray images were selected under stringent conditions; for example, appropriate acquisition parameters were considered when cases involved underweight, normal, and overweight patients. Cases with other systematic diseases were also omitted in this study. Under these conditions the consultant pulmonologist finally determined the ROI which can be regarded as the ground truth.

The improved discrimination results using PC features are possible because of its properties of using Log Gabor as its filter function of having no DC components and its ability to preserve image details relative to the Gabor features [25, 39, 40]. The only disadvantage or limitation of using PC method is that too many parameters that need to be chosen to suit the application. In this study a simulation was carried out to get the best PC parameter that yields the highest PSNR.

A simulation study was carried out which strongly suggests that the PC parameters, where *n* = 5, *o* = 6, *λ*_min⁡_ = 8, *α* = 3, *σ* = 0.55, *d* = 1, *γ* = 50, *c* = 0.2, and *k* = 2, yielded the highest PSNR (9.1575) and therefore provided evidence of discriminatory properties. A further motivation in the use of PC is that it provides local information and the texture measures of the PC in turn may be regarded as providing global information of the ROI.

To enable reasonable comparison between PC and Gabor, in this study we use the same number of channels and similar channel characteristics which are 30 channels (*n* = 5, *o* = 6) and bandwidth is 2 octaves following [42].

Five of the texture measures from PC and from Gabor were shown to have univariate normal probability distributions. An optimal discrimination procedure which judiciously selects the LDF or QDF was developed to perform pairwise discrimination. For the case *C* = 1 (total ignorance) smaller (*δ*, *β*) errors for discriminating LC and NL as well as discriminating PNEU and NL were shown if energy, contrast, and homogeneity from PC were used relative to Gabor. All texture measures performed badly for the LC and PNEU discrimination case. This prompted the use of the ROC analysis where plots of the TPF versus FPF for all *C* values aided in the selection of texture measures in more than one possible way. Firstly, the ROC curves furthest to the left and which is highest indicate the best choice of texture measures. Secondly, for a fixed low value of FPF (0.2), the texture measure with the highest TPF should be selected. Thirdly, using the area under the ROC curves shown in Table 7 yielded energy (99.86%), contrast (99.06%), and homogeneity (99.86%) from PC and gave the best discrimination results for LC-NL and PNEU-NL discriminations. For LC-PNEU discrimination, entropy (80.02%) and homogeneity (80.16%) gave the best discrimination. Our results on energy and contrast from PC verify the invariance property of PC with respect to image brightness and contrast [25].

A comparison of the use of PC and that of our earlier work [9] showed that Type I Errors and Type II Errors were much less using PC when energy, contrast, and homogeneity were used as features for discrimination.

The proposed method if followed closely as shown above should be applicable to all or most images produced or prepared by a qualified radiologist. As long as the normalization of the image is carried out, the method is robust and will yield high success rate if the discrimination is between disease absent and disease present in a large cohort study. However, for the discrimination between diseases, even with the assistance of a qualified radiologist, the proposed method is only applicable for the situation when both LC and PNEU are in their developed stage and each patient exhibits only one category of disease. These conditions must be adhered to when carrying out a large cohort study where images include other lung diseases.

## 8. Conclusion

A novel procedure for the pairwise discrimination of LC, PNEU, and NL using texture measures calculated from phase congruency values is proposed in this study. When the set of the phase congruency parameters {*n* = 5, *o* = 6, *λ*_min⁡_ = 8, *α* = 3, *σ* = 0.55, *d* = 1, *γ* = 50, *c* = 0.2, and *k* = 2} was used, good discrimination was achieved. The ROC analysis was the method used to indicate the best texture measures for the three pairwise discrimination cases. Given that the texture measures are normally distributed, energy and homogeneity from PC gave better discrimination results for LC-NL and PNEU-NL discriminations, and for LC-PNEU discrimination, entropy and homogeneity gave the best discrimination relative to Gabor. A promising result of this study is that PC texture measures have a better chance of solving the LC-PNEU discrimination problem.

## Figures and Tables

**(a):** 

	NL (40 images)				
	*C*7	*T*1	*T*2	*T*3	*T*4
Mean	3260.50	3230.40	3168.50	3207.20	3220.60
STD	104.24	170.40	178.43	162.38	162.23
Max	3393	3439	3411	3408	3435
Min	3035	2885	2884	2891	2953
Range	358	554	527	517	482

**(b):** 

	LC (50 images)				
	*C*7	*T*1	*T*2	*T*3	*T*4
Mean	3346.20	3320.80	3314.80	3377.80	3441.10
STD	207.00	239.19	235.75	220.47	176.67
Max	3644	3667	3635	3642	3663
Min	3045	2901	2952	2895	3099
Range	599	766	683	747	564

**(c):** 

	PNEU (50 images)				
	*C*7	*T*1	*T*2	*T*3	*T*4
Mean	3350.50	3350.80	3366.30	3442.10	3491.10
STD	99.77	103.68	122.55	116.11	116.75
Max	3532	3549	3592	3594	3718
Min	3195	3241	3191	3270	3312
Range	337	308	401	324	406

**Table 2:** One- and two-dimensional line profiles of PC values (*n* = 5, *o* = 6, *λ*_min⁡_ = 8, *α* = 3, *σ* = 0.55, *d* = 1, *γ* = 50, *c* = 0.3, and *k* = 2).

Type of image	Original image	Transformed image	1D-line profile	2D profile
Normal	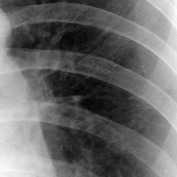	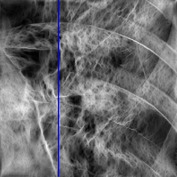	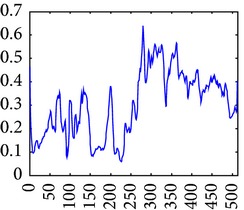	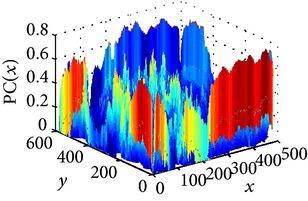

LC	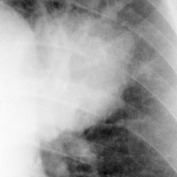	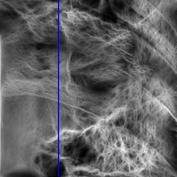	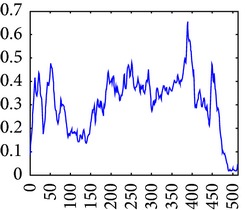	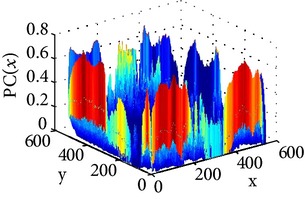

PNEU	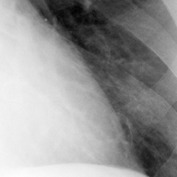	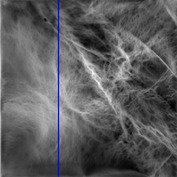	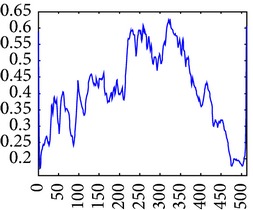	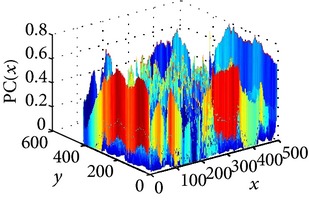

**Table 3:** Summary statistics of texture measures for NL, LC, and PNEU using PC model.

	NL		LC		PNEU	
	Mean	STD	Mean	STD	Mean	STD
*E*	34516.27	180.4126	20827.91	176.8569	21634	158.1402
$\overset{-}{E}$	0.1317	0.0012	0.0176	0.0023	0.0130	0.0019
En	7.1395	0.5147	7.0051	0.3724	6.9958	0.4145
*C*	4770335660	16368541	3656382764	23589643	3086836786	18975301
*H*	1795.1242	108.4563	1137.0325	89.7962	1009.1185	93.5230
STDV	0.0011	0.0000	0.0011	0.0000	0.0010	0.0000
STDE	0.0012	0.0000	0.0012	0.0000	0.0010	0.0000
Corr	0.9999	0.0000	0.9999	0.0000	0.9999	0.0000

**Table 4:** Summary statistics of texture measures for NL, LC, and PNEU using Gabor model using 6 orientation and 5 scales (30 channels).

	NL		LC		PNEU	
	Mean	STD	Mean	STD	Mean	STD
*E*	9144690.1780	68523.1452	460518.4185	16923.2546	687961.0539	4532.5264
$\overset{-}{E}$	79.0546	14.1895	12.7965	4.3981	16.7234	3.7526
En	0.7546	0.1521	3.0156	0.6473	2.4652	0.0198
*C*	8137060833.5314	13856471.1485	3968463851.1828	18962641.5264	4341289635.0125	21658547.4723
*H*	14482.4547	785.4213	8994.8564	352.9437	7349.9453	275.9412
STDV	0.0325	0.000	0.0756	0.000	0.0756	0.000
STDE	0.0323	0.000	0.0752	0.000	0.0752	0.000
Corr	0.9999	0.000	0.9999	0.000	0.9999	0.000

**Table 5:** Summary of testing normality for homogeneity for PC model and Gabor model.

	PC model			Gabor model		
	NL	LC	PNEU	NL	LC	PNEU
*K*-stat	0.1076	0.0839	0.1004	0.0975	0.1793	0.1186
Critical value	0.2101	0.1884	0.1884	0.2101	0.1884	0.1884
Normality	Accept	Accept	Accept	Accept	Accept	Accept

**Table 6:** Error probabilities of discrimination using LDF and QDF for *C* = 1 when PC and Gabor textures features are used in discriminant function.

Discriminated pair	PC						Gabor					
	LC and NL		PNEU and NL		LC and PNEU		LC and NL		PNEU and NL		LC and PNEU	
Texture measure	*δ*1	*β*1	*δ*2	*β*2	*δ*3	*β*3	*δ*1	*β*1	*δ*2	*β*2	*δ*3	*β*3

Energy	0.00	0.00	0.00	0.00	0.16	0.20	0.24	0.32	0.24	0.30	0.32	0.40
Mean energy	0.12	0.24	0.28	0.44	0.48	0.44	0.24	0.32	0.24	0.30	0.32	0.40
Entropy	0.16	0.32	0.44	0.48	0.56	0.36	0.08	0.12	0.16	0.20	0.28	0.28
Contrast	0.00	0.08	0.00	0.04	0.24	0.32	0.16	0.20	0.24	0.28	0.32	0.40
Homogeneity	0.00	0.00	0.00	0.00	0.16	0.28	0.12	0.16	0.20	0.24	0.28	0.24

**Table 7:** Area under the ROC curve (AUC) for each texture measure.

	LC-NL	PNEU-NL	LC-PNEU
PC			
Energy	0.9986	0.9986	0.7699
Mean energy	0.8691	0.8124	0.7547
Entropy	0.8190	0.7485	0.8002
Contrast	0.9908	0.9866	0.6988
Homogeneity	0.9986	0.9986	0.8016
Gabor			
Energy	0.6704	0.6879	0.6375
Mean energy	0.6704	0.6879	0.6375
Entropy	0.8514	0.7754	0.7458
Contrast	0.7452	0.7156	0.6415
Homogeneity	0.8291	0.7561	0.7943
